# Medical and Physical Intelligence

**Published:** 1822-08

**Authors:** 


### MEDICAL AND PHYSICAL INTELLIGENCE.

### 1. *Poisoning from Opium.*

In reference to the cases illustrating the efficacy of cold affusion in poisoning from opium, which were published in the last Number of the Repository, we have been requested by Mr. Wray to insert the following extract from the minutes of the London Medical Society:—January 29, 1821, present, Dr. Clutterbuck, president; Drs. Walsham, Blicke, and Copland; Messrs. Calloway, Andrew, Drysdale, Brown, Jarratt, Burrows, Hood, Lease, Sutliffe, Wray, and Weaver. Mr. Wray adduced instances of individuals, who, being under the influence of opium, he had roused from the state of stupor thereby induced, by dashing suddenly and repeatedly upon the head basinsful of cold water. The effect was in all these cases very remarkable: the coma was removed, and the patients were enabled to swallow emetics, by which means the poison was removed from the stomach.

### 2. Osiander's *Cabinet of Midwifery.*

"What I found very interesting at Gottingen, was Osiander's Cabinet of Midwifery, who died about a month before my arrival in that dull and stupid place. This cabinet was shown me by his son, who has succeeded to the chair of his late father. I am not aware that you are acquainted with the first part of certainly a most interesting work, published by the deceased Osiander in 1821, but which, in consequence of his death, unfortunately remains unfinished. Although, in this part, he has only been able to treat of the anatomy of the gravid uterus, and of monsters; nevertheless, under this last head, the volume seems to me to be really of great merit,—not only on account of his own numerous and valuable observations, which are truly important, but also on account of the tasteful arrangement of the most curious observations of others, scattered in the best scientific collections: in short, I found this Cabinet of Midwifery of Osiander certainly the richest I have ever seen, in regard to the collection of human monsters, and those of other animals, which it contains. A pair of forceps, invented by himself, is really particular; the branches being not, as they usually are, scooped out, but quite flat, so that each branch forms a metallic plate of the thickness of a line and one-third, and very heavy. Nevertheless, these forceps are preferred, by the professional gentlemen here, to all the others for extracting the living and dead fœtus; which is not the case in England or France. I observed, amongst the other preparations, one of a gravid uterus, in which there are distinctly seen about the neck a number of fibres interwoven with each other in various directions, after the manner of the ventricles of the heart, so that, had I seen them contracted by means of the galvanic influence, I should not have hesitated a moment in asserting that they were muscular fibres. I was also shown, as a very great curiosity, a vessel containing some uterine blood, collected at the time of the operation for an impervious hymen, and which has been preserved unaltered during a period of twenty years, although in contact with about an inch of air. Osiander was also a follower of Lavater, from what I could observe; at least, he had collected together a large quantity of masks: unfortunately, they only had the face and forehead, and no skull!—*(Private Letter.)*

### 3. *Black Urine.*

It appears from Dr. MARCET's paper in the Medico-Chirurgical Transactions, that he has met with some cases in which black urine had been voided. At the request of Dr. Marcet, some was examined by Dr. PROUT, who gives the following account of its chemical properties:

The residuum obtained from this urine by evaporation, not only does not contain any lithic acid, as was observed by Dr. Marcet, but no urea can be detected in it by the tests which indicates its presence.

Although the addition of dilute acids produced no immediate change of colour in the urine, yet, on standing for some time, a black precipitate slowly subsided, leaving the supernatant fluid transparent, and but slightly coloured.

The black precipitate thus obtained was found to be nearly insoluble either in water or alcohol, whether hot or cold. It readily dissolved in cold concentrated sulphuric and nitric acid, forming a deep brownish-black solution; but, on diluting the acids with water, the black substance appeared to be again precipitated unaltered. These acids, however, by the assistance of heat, apparently decomposed it. The black substance readily dissolved in the fixed alkalies and in the alkaline subcarbonates, forming very dark solutions. The addition of water did not affect these solutions; but acids re-precipitated the substance apparently unchanged. When ammonia was employed as the solvent, and the excess expelled by evaporation to dryness, a black or deep-brown residuum was obtained, which appeared to be a compound of the black substance with ammonia, and possessed the following properties:

It was very soluble in water; and, on being heated with caustic potash, it gave off the smell of ammonia. The black compound, however, did not appear to have any tendency to assume the crystalline form.

In evaporating to dryness, on a piece of glass, the ammoniacal solution in which the black substance had been dissolved, the residuum split into most minute fragments, having a regular and very peculiar appearance, especially when examined with a magnifier.

From the solutions of this compound in water, muriate of barytes and nitrate of silver produced copious brown precipitates, as did also proto-nitrate of mercury and nitrate of lead; but oxymuriate of mercury produced no immediate precipitate, and that obtained from acetate of zinc was of a paler brown colour.

From these experiments, Dr. Prout concludes that the remarkable specimen of urine in question owes its black colour to a compound of a peculiar principle with ammonia, as Dr. Marcet had inferred from his own trials; but he is, moreover, inclined to think that the black principle itself, such as is obtained from the urine by the action of dilute acids, may be considered as a new body possessed of acid properties. From the small quantity of the specimen, however, which could be spared for

Dr. Prout's experiments, it was impossible to obtain complete and decisive evidence on the nature of this substance; but it appears to be sufficiently characterized as a peculiar acid, and to bear a closer analogy to the lithic acid, or rather to some of the compounds which it forms when acted upon by the nitric acid, than to any other principle usually found in the urine.

Should this view of the subject be confirmed by further observations, Dr. Prout would propose to distinguish this new substance, on account of its black colour, by the name of *Melanic acid.—(Annals of Phil.)*

4. *Acetate of Morphine.*

M. ALLONEAU, M.D. of Thouars, has detailed eight cases in which he has given this remedy to adults: it was given in doses of a quarter of a grain to one grain per diem, beginning sometimes with a quarter, sometimes with half a grain. Of these eight patients, five were consumptive: the remedy was given to allay the cough and to procure sleep. In two of these cases it succeeded, in the three others it failed. The other three patients had chronic diseases of the ovary, the heart, and the stomach. This last patient was entirely cured. The affection of the heart was relieved by it; but the effect was only temporary. In the case of diseased ovary, it cured all the numerous sympathetic irritations that existed; but the complaint remained unchanged. It must be remarked, that Dr. MAGENDIE had before employed this medicine to relieve the pain of a schirrous breast. The author confirms the observation of Mr. LENS, that morphine in these doses does not cause excitement, head-ach, constipation, or stupor, as the other preparations of opium do.—*(Bibl. Med.* Febr.)

5. *Corrosive Sublimate.*

Dr. DAVY has made some interesting observations on corrosive sublimate. It is known that the liquor hydrargyri oxymuriatis of the London Pharmacopœia, on exposure to light, slowly undergoes decomposition; and it has been asserted that light has a similar effect on corrosive sublimate itself. Dr. Davy relates a number of experiments made to investigate these points. He finds that corrosive sublimate remains unaltered on exposure to light; that it remains unaltered when exposed in solution in media having a strong affinity for it, as alcohol, ether, muriatic acid, &c.; and that decomposition takes place only under circumstances of complicated affinities, as in the instance of the liquor hydrargyri oxymuriatis, and in the aqueous solution, when calomel and muriatic acid appear to be formed, and oxygen evolved.

For the purpose of further illustration of the subject, Dr. Davy describes a series of experiments on corrosive sublimate with alcohol, ether, several oils, muriatic and the mineral acids, many of the muriates, &c.; the results of which hardly admit of being given in the form of abstract. In every instance that an oil, whether volatile or fixed, was heated with corrosive sublimate, mutual decomposition took place, charcoal was evolved, and muriatic acid and calomel formed. Besides, when oil of turpentine was used, some traces of artificial camphor appeared; and, when the oils of cloves and peppermint, a purple compound distilled over, consisting of the oil employed and muriatic acid. With muriatic acid, common salt, and some other muriates, corrosive sublimate formed definite compounds remarkable for their solubility. —*(Annals of Philosophy.)*

### 6. *New Test for Arsenic.*

Dr. COOPER, president of Columbia College, finds a solution of chromate of potash to be one of the best tests of arsenic. One drop is turned green by the fourth of a grain of arsenic, by two or three drops of Fowler's mineral solution, or any other arsenite of potash. The arsenious acid takes oxygen from the chromic, which is converted into green oxide. To exhibit the effect, take, he says, five watch-glasses; put on one, two or three drops of a (watery) solution of white arsenic; on the second, as much arsenite of potash; on the third, one-fourth of a grain of white arsenic in the substance; on the fourth, two or three drops of solution of corrosive sublimate either in water or alcohol; in the fifth, two or three drops of a solution of copper. Add to each three or four drops of solution of chromate of potash. In half an hour, a bright, clear, grass-green colour will appear in Nos. 1, 2, 3, unchangeable by ammonia; No. 4 will instantly exhibit an orange precipitate; No. 5 a green, which a drop of ammonia will instantly change to blue. Dr. Cooper, however, does not recommend that this test should be exclusively relied on, but merely that it should be used in conjunction with others, of which the most unequivocal is certainly the actual exhibition of arsenic in a metallic form.—*(Silliman's Journal.)*

### 7. *Explosion.*

It is lamentable to think that, although the means of preventing explosions in mines are so simple and easily procured as to be within the reach of all, there should still be instances of these dreadful occurences. —On Monday last, a dreadful accident occurred at the Intake Colliery, near this town. Ten men and boys descended into the pit early in the morning: the noxious gas, called by the miners *fire-damp*, coming in contact with the lighted candle, ignited, and a terrible explosion ensued, which shivered many of the supporters of the roof of the mine to atoms, and brought down the incumbent mass in one tremendous crash. The explosion took place at a part of the mine remote from the pit, where two men and one boy were. Two others, who heard the noise, and saw the instantaneous burst of fire, shrunk into one of the stables where the horses are kept: the flame rushed past them with terrible velocity, ascended the shaft, and blazed in one immense volume of fire from the mouth of the pit. Some men, who were near, describe the appearance, the thunder, and the crash, as tremendously awful and appalling. When the flame had passed away from out the mine, the men who had taken shelter in the stable ran to the corves by which they had descended: one man was burning as they passed him; he was alive, and called to them for assistance; they placed him, and a boy almost senseless, in the corve, and the four were drawn out of the pit alive. The two who had been exposed to the fire soon after expired; and, of the ten who descended into the mine in the morning, only two are now alive.—*(Sheffield Independent, July 6th.)*

### 8. *Analysis of an Aerolite.*

M. LAUGIER states that he has performed four analyses of the aerolite which fell at Juvenas: the first by means of acid; the second by potash; the third by nitric acid, with the intention of determining the quantity of sulphur; the fourth by means of nitrate of barytes, for the purpose of determining the quantity of the potash which M. Vauquelin had found in this stone, although he did not employ this method, the only one which can be relied upon. These several analyses, all agreeing as to the nature of the elements of stone, varied slightly with respect to their proportions; a variation which must be attributed to its being deficient in homogeniety in all its parts.

The second analysis, that by potash, which appeared to M. Laugier to be the most correct, gave the following results:

| | |
|---|---|
| Silica | 40 |
| Oxide of iron | 23.5 |
| Oxide of Manganese | 6.5 |
| Alumina | 10.4 |
| Lime | 9.2 |
| Chrome | 1 |
| Magnesia | 0.8 |
| Sulphur | 0.5 |
| Potash | 0.2 |
| Copper | 0.1 |
| Indispensable loss | 3.0 |
| Loss from unknown causes | 4.8 |
| | 100.0 |

M. Laugier observes, that the loss of four or five per cent. which always occurred in his analyses, instead of the increase which, in these kinds of analyses, usually results from metals which the aerolites contain, renders it probable that, in the aerolite of Juvinas, the iron and manganese exist in the state of oxides. No portion of this aerolite reduced to powder was attracted by the magnet, which renders this conjecture more probable.

M. Laugier endeavoured to discover whether this loss was owing to carbonic acid, but the stone did not appear to contain any: in a subsequent analysis he found, however, that it yielded rather more sulphur than stated in the analysis. He afterwards observes, that this aerolite resembles one which fell at Ionzac in its analysis, and especially in the absence of nickel; and also with an aerolite which fell, in 1813, in the environs of Lantola, a village in the government of Wibourg, in Finland. These are the only aerolites which have been hitherto found destitute of nickel.—*(Annals of Philosophy.)*

### 9. *Meteoric Iron.*

Dr. WILLIAM ZIMMERMAN, professor of chemistry in the university of Giessen, has discovered that all the aqueous atmospherical precipitates or deposits, (dew, snow, rain, and hail,) during that period, contained meteoric iron, which was usually combined, in the same manner as in meteoric stones, with nickel. Almost all the rains contained common salt, and a new organic substance composed of hydrogen, oxygen, and carbon, which the discoverer has called *pyrine*. In the same manner the rain water was found, on several occasions, indubitably to contain several kinds of earths. The rains in February and March particularly abounded in these ingredients, which are found also in the meteoric stones. From contemporary observations made on various eminences, Diensberg, the Castle of Gleiberg, a tower of the barracks at Giessen, &c. various other results were obtained, several of which are in favour of the opinion that the stony meteoric masses are of telluric, and not of cosmic, origin.—*(New Monthly Mag.)*

### 10. *Musty Flour.*

E. Davy, Esq. of the Cork Institution, has discovered that the carbonate of magnesia, in small quantity, has the property of restoring to its primitive state flour which has a musty smell and taste, from dampness and other causes. "One pound of the carbonate of magnesia (common magnesia of the shops,) is to be combined with 250lbs. of musty flour; that is, in the minor proportion of thirty grains of the carbonate to one pound of flour. It is to be leavened and baked in the usual way of making bread. The loaves will be found to rise well in the oven, to be more light and spongy, and also whiter, than bread in the common way. It will likewise have an excellent taste."

### 11. *Congenital Luxation of the Tibia.*

Mr. A. C. Chatelain, of Neuveville, Switzerland, has published a case of this uncommon accident. The child, who was the subject of it, was born on the 6th of April, 1820, after a natural labour; and Mr. Chatelain was called in on the day following, in consequence of the leg being constantly kept bent upon the anterior part of the thigh: it was evident that the tibia was luxated backwards. It was reduced by the gentlest extension; but, the moment it was left to itself, it immediately resumed its former position, by the mere action of the extensor muscles. To maintain the reduction, Mr. Chatelain applied a bandage composed of three splints of tin, covered with sheep's leather, and fixed to two little straps placed half an inch from each extremity, so that one of the splints commanded the posterior surface of the knee, and the two others the lateral surfaces. The cure was perfected by the twenty-third day; but not until the leg had been kept permanently bent upon the posterior part of the thigh, in order to deprive the extensor muscles of the action they seemed to have acquired at the expence of their antagonists. No trace of the disease remained, and the girl has walked for some months as well as any child of her age. Mr. Chatelain is at a loss to account for this accident, which, from the total want of power in the flexors of the thigh, he apprehends to have existed long prior to the accouchement. The mother experienced a violent fall in the seventh month of her pregnancy.—*(Bibliotheque Medicale, January.)*

12. *New Muscle.*

Dr. HORNER, one of the professors of anatomy in Philadelphia, has lately had occasion to observe, in dissecting the human eye, a part of its muscular apparatus, which he believes is not generally known. The part alluded to is a small oblong muscle, found on the posterior part of the lachrymal ducts. It arises from the os unguis near its junction with the os planum, and, passing forwards and outwards, terminates at the internal commissure of the eyelids, near the puncta lachrymalia. As it approaches the eyelids, it splits into two parts; one is inserted into the upper lid, and the other into the lower; the superior fibres of the first named part, or the upper, being blended, in some measure, with the orbicularis muscle, but the lower having fully a distinct and well-marked insertion. The muscle is about half an inch long, and one-fourth of an inch wide, its upper and lower margins being well defined. Dr. Horner has also had occasion to make some new remarks on a tendinous structure enveloping the lachrymal ducts, and the position of these ducts, by which the curvature of the internal commissure of the eyelids is kept up, and which would prevent any deformity of the lids, even if the tendon of the orbicularis were cut through in the operation for fistula lachrymalis. The origin of the muscle is at least half an inch posterior to the orbicularis tendon, at its attachment to the nasal process, and it lies on the tendinous matter including the ducts. To get a view of this muscle, cut through the eyelids, and separate them from the ball, except at the internal canthus; then turn the lids over on the nose; remove the valvula semilunaris and the tunica conjunctiva in the neighbourhood, with the fatty matter, and you will be sensible of the arrangement described. Its use is to draw in the puncta, and to keep the edges of the eyelid properly adjusted to the ball, &c.

*(London Medical Repository.)*

13. *Croup.*

M. VILLENEUVE relates the following fact. He was called to a child attacked by angina tonsillaris, which appeared to be very slight. Two days afterwards he saw the child, and, to his great astonishment, found all the most acute symptoms of the croup present. All curative means proved unavailing, and the child died. M. Villeneuve, having before seen a case in which an attack of angina tonsillaris, in a child of four years of age, disguised an attack of croup equally fatal, urges the necessity of paying the most scrupulous attention to the state of the organs of respiration, whenever the least change is perceived in the voice of children, although the change may appear to be the result of a different affection.—*(Bibl. Med.* April.)

14. *Croup.*

Dr. REDDELIN, of Wismar, has communicated to the Royal Society of Gottingen, through Professor BLUMENBACH, the following successful treatment of croup, after the usual remedies had been tried without effect. The patient was a female, aged nineteen, who, on the third day after being seized with the croup, was unable to swallow, had begun to rattle in the throat, and seemed approaching rapidly her dissolution. Dr. Reddelin insinuated, by means of a quill, a mixture of Spanish snuff and marocco into her nostrils, and, after repeating this mixture a second time, it excited sneezing and vomiting: this occasioned the discharge of two long membranous cylinders from the trachea, upon which the rattling immediately ceased, and the patient rescued from instantaneous suffocation. One of the tubes, when slit open, measured nine French lines in breadth; they were quite elastic, and bore a strong extension without injury to their fibrous texture.—*(New Mon. Mag.)*

### 15. *Bronchocele.*

We have been informed, by a correspondent from Horsham, Sussex, that the iodine has been found very successful in the cure of bronchocele in that town and its vicinity. Ten drops of the tincture of iodine taken three times a-day, has generally been found to remove the complaint in the course of five or six weeks. The subjects are almost all females, about the age of puberty. In our next we hope to be able to give a more detailed account of some of these cases. It must be remarked, that bronchocele is a disease of very frequent occurrence in that part of the country, and that it scarcely ever is met with in the male sex.

### 16. *External Application of Stramonium in Chronic Rheumatism.*

Stramonium, in the United States, is employed in the form of an ointment in cases of chronic rheumatism. Dr. Zollickoffer says, he has known it to succeed when the internal exhibition of the medicine seemed to have no effect. He employs a tincture of the plant, made in the proportion of one ounce to half a pint of proof spirit, instead of the extract; the latter of which we consider to be a very unnecessary addition to the Pharmacopœia.

### 17. *College of Physicians.*

The Crown has bestowed a piece of ground in Pall Mall, East, on which is to be erected a new edifice for the College of Physicians. His Majesty has likewise ordained that the president shall hereafter be, *ex officio*, one of his physicians in ordinary.

### 18. *Small-Pox Inoculation.*

On Thursday, June 6th, at a general meeting of the governors of the Small-Pox Hospital, it was unanimously resolved to discontinue the practice of variolous inoculation at that hospital.

### METEOROLOGICAL JOURNAL.

By Messrs. WILLIAM HARRIS and Co. 50, Holborn, London.

*From June* 20, 1822, *to July* 19, 1822.

| | MOON. | Rain gauge | THERM. | | | BAROM. | | DE LUC'S HYG. | | WIND. | | ATMOSPHERIC VARIATION. | | |
|---|---|---|---|---|---|---|---|---|---|---|---|---|---|---|
| | | | 9 A.M. | MAX. | MIN. | 9 A.M. | 10P.M. | 9 A.M. | 10P.M. | 9 A.M. | 10P.M. | 9 A.M. | 2 P.M. | 10P.M |
| June | | | | | | | | | | | | | | |
| 20 | | | 55 | 67 | 53 | 29.94 | 29.97 | 61 | 58 | ESE | E | Overcast | | |
| 21 | | | 56 | 66 | 52 | 30.00 | 30 08 | 55 | 56 | ENE | SE | Fine | | |
| 22 | | .01 | 62 | 75 | 53 | 30.00 | 29.96 | 56 | 50 | E | SE | Fine | Sho'ry | Fine |
| 23 | | .05 | 66 | 78 | 63 | 29.90 | 29.88 | 62 | 69 | SW | W | Rain | Fine | |
| 24 | | | 68 | 76 | 62 | 29.90 | 29.92 | 58 | 40 | NW | NW | Fine | | |
| 25 | | | 70 | 77 | 62 | 30.00 | 29.98 | 59 | 48 | WNW | W | Fine | | |
| 26 | ☾ | .03 | 76 | 82 | 63 | 29.96 | 29.88 | 51 | 52 | WNW | W | Fine | | Sho'ry |
| 27 | | | 70 | 74 | 58 | 29.95 | 30.06 | 47 | 40 | N | NE | Cloudy | Fine | |
| 28 | | .18 | 68 | 74 | 58 | 30.03 | 29.86 | 51 | 70 | W | WSW | Cloudy | | Rain |
| 29 | | .05 | 61 | 67 | 58 | 29.85 | 29.96 | 62 | 53 | NNW | NNE | Cloudy | Rain | Cloud |
| 30 | | | 65 | 72 | 55 | 29.90 | 29.84 | 55 | 57 | W | NW | Cloudy | Sho'ry | Cloud. |
| July | | | | | | | | | | | | | | |
| 1 | | | 62 | 80 | 60 | 29.92 | 29.94 | 51 | 50 | N | NW | Fine | | |
| 2 | | | 62 | 70 | 54 | 29.92 | 29.73 | 57 | 50 | W | W | Cloudy | | |
| 3 | | | 64 | 72 | 50 | 29.80 | 29.87 | 50 | 48 | N | W | Fine | | |
| 4 | ○ | | 66 | 75 | 58 | 29.84 | 29.80 | 58 | 58 | WSW | WSW | Cloudy | Fine | |
| 5 | | .75 | 62 | 66 | 58 | 29.64 | 29.58 | 70 | 60 | SE | NW | Rain | Cloud. | |
| 6 | | | 64 | 70 | 55 | 29.75 | 29.90 | 52 | 48 | ENE | SE | Cloudy | | |
| 7 | | | 66 | 72 | 60 | 30.00 | 29.30 | 48 | 54 | NW | N | Fine | Cloud. | |
| 8 | | | 64 | 72 | 62 | 30.02 | 30.00 | 58 | 56 | N | NNE | Cloudy | | |
| 9 | | .13 | 64 | 69 | 60 | 29.95 | 29 84 | 61 | 62 | WNW | W | Overcast | Rain | Cloud. |
| 10 | | .07 | 63 | 72 | 57 | 29.74 | 29.74 | 66 | 55 | WSW | NW | Showery | Fine | |
| 11 | ☽ | .15 | 61 | 73 | 60 | 29.72 | 29.44 | 61 | 69 | NW | WSW | Fair | Sho'ry | Rain |
| 12 | | | 66 | 71 | 55 | 29.29 | 29.62 | 57 | 60 | NW | N | Cloudy | Sho'ry | Cloud |
| 13 | | | 62 | 70 | 56 | 29.77 | 29.88 | 54 | 51 | N | E | Fine | | |
| 14 | | | 60 | 75 | 57 | 29.90 | 29.91 | 50 | 55 | E | SSE | Fine | | |
| 15 | | | 61 | 76 | 62 | 29.83 | 29.74 | 56 | 60 | ESE | E | Overcast | Fine | Sho'ry |
| 16 | | | 64 | 73 | 60 | 29.90 | 30.00 | 56 | 50 | E | ENE | Fine | | |
| 17 | | .31 | 65 | 70 | 58 | 29.91 | 29.61 | 55 | 61 | W | SW | Showery | | Rain |
| 18 | ● | | 66 | 78 | 61 | 29.70 | 29.55 | 63 | 60 | S | SE | Fine | Sho'ry | Fair |
| 19 | | .09 | 67 | 79 | 63 | 29.34 | 29.41 | 60 | 59 | S | SW | Showery | Fine | |

The quantity of rain during the month of June, was 1 inch and 11-100ths.

